# Antifungal activity, kinetics and molecular mechanism of action of garlic oil against *Candida albicans*

**DOI:** 10.1038/srep22805

**Published:** 2016-03-07

**Authors:** Wen-Ru Li, Qing-Shan Shi, Huan-Qin Dai, Qing Liang, Xiao-Bao Xie, Xiao-Mo Huang, Guang-Ze Zhao, Li-Xin Zhang

**Affiliations:** 1 State Key Laboratory of Applied Microbiology Southern China, Guangdong Provincial Key Laboratory of Microbial Culture Collection and Application, Guangdong Institute of Microbiology, Guangzhou, 510070, China; 2 Institute of Microbiology, Chinese Academy of Sciences (CAS), Beijing, 100101, China

## Abstract

The antifungal activity, kinetics, and molecular mechanism of action of garlic oil against *Candida albicans* were investigated in this study using multiple methods. Using the poisoned food technique, we determined that the minimum inhibitory concentration of garlic oil was 0.35 μg/mL. Observation by transmission electron microscopy indicated that garlic oil could penetrate the cellular membrane of *C. albicans* as well as the membranes of organelles such as the mitochondria, resulting in organelle destruction and ultimately cell death. RNA sequencing analysis showed that garlic oil induced differential expression of critical genes including those involved in oxidation-reduction processes, pathogenesis, and cellular response to drugs and starvation. Moreover, the differentially expressed genes were mainly clustered in 19 KEGG pathways, representing vital cellular processes such as oxidative phosphorylation, the spliceosome, the cell cycle, and protein processing in the endoplasmic reticulum. In addition, four upregulated proteins selected after two-dimensional fluorescence difference in gel electrophoresis (2D-DIGE) analysis were identified with high probability by mass spectrometry as putative cytoplasmic adenylate kinase, pyruvate decarboxylase, hexokinase, and heat shock proteins. This is suggestive of a *C. albicans* stress responses to garlic oil treatment. On the other hand, a large number of proteins were downregulated, leading to significant disruption of the normal metabolism and physical functions of *C. albicans*.

Currently, disseminated invasive candidiasis has an estimated mortality rate of 40%, even with the use of antifungal drugs^1^. *Candida albicans* is the primary cause of candidiasis and is the fourth most common cause of nosocomial infection^2^. *C. albicans* is an opportunistic pathogen of humans and an endogenous member of the human microbiota. In the past two decades, infections caused by *C. albicans* have increased significantly^3^. A characteristic feature of *C. albicans* is its ability to grow either as unicellular budding yeast or in filamentous form^1^. Moreover, *C. albicans* growing on medical implants, such as blood and urinary catheters or heart valves, frequently self-organizes into biofilms composed of a dense network of yeasts, hyphae, pseudohyphae and a self-produced matrix of extracellular polymeric material^4^,^5^,^6^. *C. albicans* biofilms are resistant to a variety of antifungal drugs, making conventional antifungal agents ineffective for the treatment of *C. albicans* infections^7^. For example, *C. albicans* cells in biofilms are 100 times or more resistant to fluconazole and 20 times or more resistant to amphotericin B than those in the planktonic form^8^. Besides being pathogenic, *C. albicans* also provides an excellent eukaryotic model system to explore the antifungal mechanisms of potent drugs^9^.

Garlic is a common food that has been widely used in traditional medicine for thousands of years^10^. Garlic oil, which is extracted from garlic, has been shown to have effective antifungal and anti-inflammatory activities^11^,^12^,^13^,^14^,^15^. Diallyl trisulfide (DTS) and diallyl disulfide (DDS) are the most abundant volatile sulfur-containing compounds in garlic oil^16^,^17^. Our experiments showed that garlic oil had excellent antifungal activity against *C. albicans*. Therefore, the antifungal activity, kinetics, and molecular mechanism of action of garlic oil against *C. albicans* ATCC 10231 were studied using multiple techniques. Our study provided new knowledge regarding the antifungal effect of garlic oil.

## Results

### The antifungal activity of garlic oil against *C. albicans*

**Figure 1:**
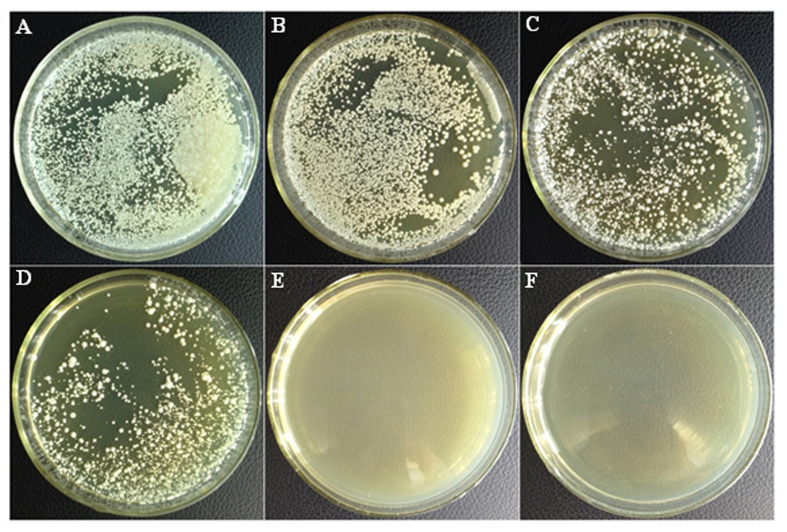
Petri dish photos of antifungal activity assay of garlic oil with different concentrations (μg/mL) against *C. albicans* in 7-day incubation using the poisoned food technique. (**A**) Control, (**B**) 0.04, (**C**) 0.09, (**D**) 0.17, (**E**) 0.35, (**F**) 0.69.

In order to determine the antifungal activity of garlic oil, *C. albicans* cells were treated with different concentrations of garlic oil by poisoned food technique. The experimental results are shown in Fig. 1. After one day of incubation, the control petri dishes were covered with white colonies, whereas no colonies were observed in the other five experimental groups exposed to garlic oil. However, after seven days of incubation, the petri dishes of the control group, as well as the 0.04, 0.09, and 0.17 μg/mL garlic oil groups were fully covered with white colonies of *C. albicans*. Moreover, the colonies observed in the 0.04, 0.09, and 0.17 μg/mL garlic oil petri dishes were all covered with white colonies despite having more than a three-day delay in their growth compared to the control. No colonies were identified on the 0.35 μg/mL and beyond garlic oil-treated petri dishes during the seven-day incubation. Therefore, the minimum inhibitory concentration (MIC) of garlic oil against *C. albicans* was determined to be 0.35 μg/mL.

### Fungicidal kinetics of garlic oil against *C. albicans*

**Figure 2:**
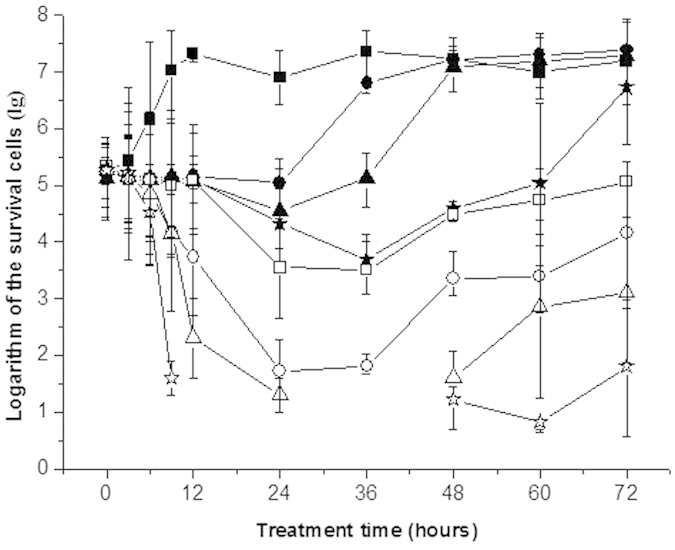
Logarithmic fungicidal kinetic curves of garlic oil against *C. albicans*. The concentrations (μg/mL) of garlic oil were 0 (▄), 0.04 (●), 0.09 (▲), 0.17 (★), 0.35 (◻), 0.69 (○), 1.39 (△) and 2.77 (✰).

The fungicidal kinetic curves of garlic oil against *C. albicans* are shown in Fig. 2. The quantities of the surviving cells in all experimental groups were calculated based on the numbers of fungal colonies that grew on the petri dishes. The initial cell concentration in every experimental group was 10^5^ colony-forming units (CFU)/mL. The fungicidal kinetic curves showed that *C. albicans* in the control group grew to exponential phase after a 3-h lag phase and reached the stabilization phase after incubation for 12 h. In contrast, a 24-h growth delay and a slight decline in the number of surviving cells were determined in the 0.04 and 0.09 μg/mL garlic oil groups. The cells reached exponential phase and stabilization phase after incubation for 24 h and 48 h, respectively. More than 90% of cells were killed after treatment for 24 h in the 0.17 and 0.35 μg/mL garlic oil groups, and after 9 h in the 0.69, 1.39 and 2.77 μg/mL garlic oil groups. A small number of persistent cells began to grow again gradually after 2 or 3 days of incubation. In addition, a trend was observed that with increasing concentrations of garlic oil, the rate of cell killing and the duration of growth lag phase increased correspondingly. These data indicated that garlic oil had a time- and dose-dependent antifungal effect against *C. albicans*.

### The internal morphology of *C. albicans* observed by transmission electron microscopy (TEM)

**Figure 3:**
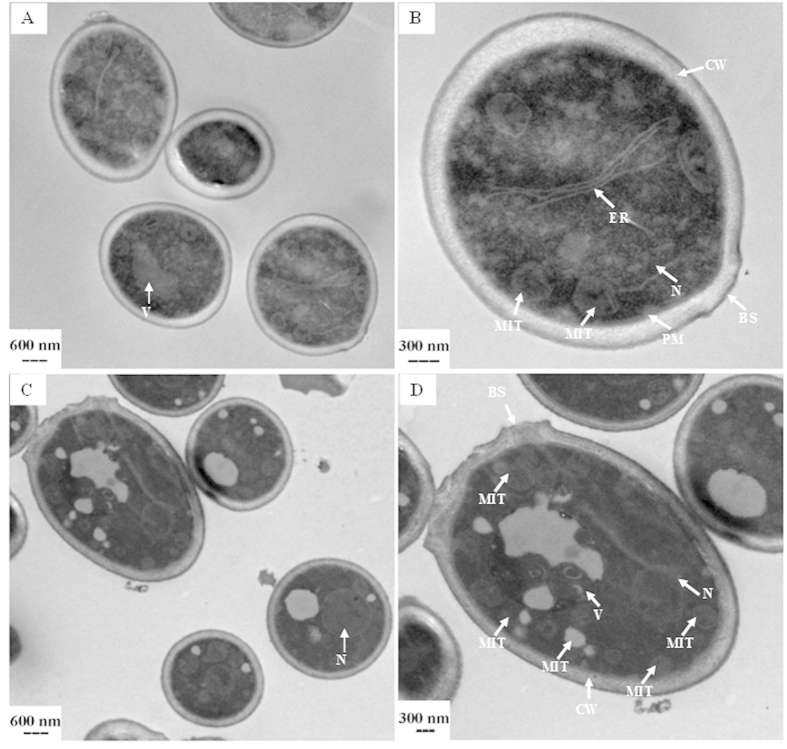
Internal morphological changes of *C. albicans* observed by TEM. (**A**,**B**) control; (**C**,**D**) treatment with 1.39 μg/mL garlic oil for 2 h. BS: bud scar; CW: cell wall; ER: endoplasmic reticulum; MIT: mitochondria; N: nucleus; PM: plasma membrane; V: vacuoles.

The photographs of the internal morphology of garlic oil-treated *C. albicans* cells observed by TEM are shown in Fig. 3. The regular internal structure of *C. albicans* can be observed in the cells of the control treatment (Fig. 3A,B). The cellular organelles, such as the cellular wall, plasma membrane, mitochondria, endoplasmic reticulum, nucleus and vacuole, were all clearly visible. In contrast, dramatic changes were observed in the *C. albicans* cells treated for 2 h with 1.39 μg/mL garlic oil. Some cellular organelles were severely damaged, such as vacuoles, mitochondria, and storage granules. The nucleus and cellular wall showed no apparent damage except for the destroyed bud scar (Fig. 3C,D).

### RNA sequencing results of *C. albicans* after exposure to garlic oil

The RNA sequencing results revealed that a large number of genes in *C. albicans* were differentially expressed after garlic oil treatment. The volcano plots of the differentially expressed genes (Fig. S-1) demonstrated that nearly three thousand genes were differentially expressed, with either an increase or decrease of more than two-fold (the spots marked in red). Most genes were downregulated (left side of the y-axis), while a small fraction was upregulated (right side of the y-axis).

**Figure 4:**
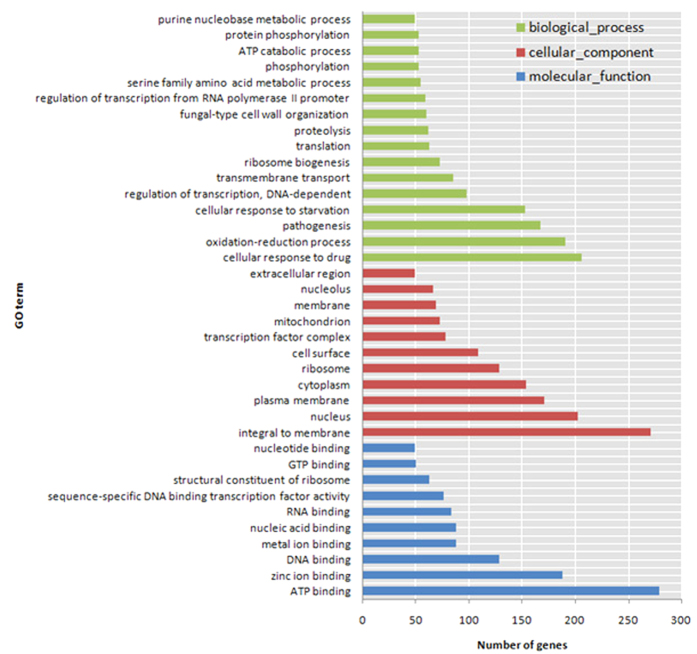
Significantly enriched Gene Ontology categories (*q* value < 0.05) with differentially expressed genes in the treatment group compared to the control group. The results can be summarized in three main categories: biological process, cellular component and molecular function.

To investigate changes in the patterns of gene expression of *C. albicans* after garlic oil treatment, the percentage of genes in each GO category was analyzed. The GO categories significantly enriched (*q* < 0.05) among those differentially expressed genes are shown in Fig. 4. Within the biological process category, 16 terms were enriched in differentially expressed genes. Among them, cellular response to drugs, oxidation-reduction processes, pathogenesis, and cellular response to starvation were significantly enriched. The ATP-binding, zinc-ion-binding and DNA-binding terms were significantly enriched in the molecular function category. Membrane-integrated, nucleus, plasma membrane, cytoplasm, ribosome, and cell surface terms were significantly enriched in the cellular component category.

To further understand the biological functions of the differentially expressed genes, KEGG analysis was performed to classify the functions of the identified genes. The differentially expressed genes were mapped to 19 pathways in the KEGG database, as shown in Table S-1. These 19 KEGG pathways were all essential to the survival and reproduction of *C. albicans*, including oxidative phosphorylation, spliceosome, cell cycle, protein processing in the endoplasmic reticulum, pyrimidine metabolism, meiosis, RNA transport, ribosome biogenesis, RNA degradation, proteasome, the mRNA surveillance pathway, nucleotide excision repair, basal transcription factors, DNA replication, RNA polymerase, various types of N-glycan biosynthesis, protein export and the MAPK signaling pathway.

### 2D-DIGE proteomic results of *C. albicans* exposed to garlic oil

**Figure 5:**
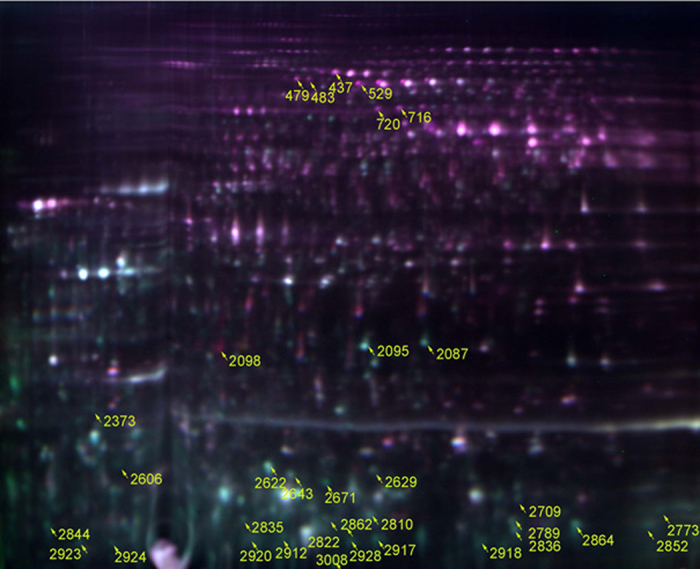
Two-dimensional DIGE gel images of the *C. albicans* proteome. Proteins were separated in the first dimension at pH 3.0–10.0 and on a 12% SDS–PAGE gel in the second dimension. Control (green fluorescence) and 1.39 μg/mL (red fluorescence) of garlic oil group.

A photo of the 2D-DIGE analysis gel is shown in Fig. 5. A merged image of protein samples of pool (Cy2−), control (Cy3−) and 1.39 μg/mL garlic oil groups (Cy5−) labeled by blue, green and red fluorescence, respectively, is shown in Fig. 5. Comparison of the proteome of *C. albicans* treated with garlic oil and that of the control treatment, suggested that most differentially expressed proteins were downregulated, while only a small number of proteins were upregulated. Four upregulated proteins and five downregulated proteins were selected for mass spectrometry (MS) analysis. The four upregulated proteins were identified with high probability as putative cytoplasmic adenylate kinase (5.13-fold upregulation), pyruvate decarboxylase (4.41-fold upregulation), hexokinase (3.19-fold upregulation), and heat shock protein Ssc1 (3.13-fold upregulation). The five downregulated proteins were identified with high probability as hypothetical proteins CaO19.11218, CaO19.2924, CaO19.1102, CaO19.3622, and CaO19.4311. Details of the nine identified proteins are shown in Table 1.

## Discussion

The results of antifungal activity measurement by the poisoned food technique demonstrated an MIC of garlic oil against *C. albicans* of 0.35 μg/mL. Compared to our previous study, this study indicated that garlic oil has stronger antifungal activity against *C. albicans* than *Penicillium funiculosum*, against which the garlic oil has an MIC of 0.69 μg/mL^17^. The antifungal kinetic curves of garlic oil against *C. albicans* indicated that garlic oil has a time- and dose-dependent antifungal effect. Due to the gradual evaporation and consumption of garlic oil in broth cultures, a small number of persistent *C. albicans* cells were able to grow after an initial lag phase, the duration of which was correlated to the initial garlic oil concentration.

Essential oils can penetrate the plasma membrane because of their lipophilic characteristic^18^,^19^. TEM observation showed that some organelles, such as the mitochondria and vacuoles, were damaged in C. albicans cells after garlic oil treatment. These observations were consistent with the damages observed in *P. funiculosum* mycelia treated with garlic oil^17^ and *Aspergillus niger* treated with citronella oil^20^, and indicated that garlic oil could penetrate the membranes of organelles such as the mitochondria.

The volcano plots showed that nearly three thousand genes were differentially expressed, and most of them were downregulated. The gene expression of *C. albicans* was severely altered by garlic oil treatment. GO analysis demonstrated that over 200 differentially expressed genes were enriched in the GO term of cellular response to drugs. Thus, *C. albicans* cells engaged in drug response following treatment with garlic oil. This observation is consistent with the high antifungal activity of garlic oil against *C. albicans*. Nearly two hundred differentially expressed genes were involved in the oxidation-reduction processes. This result suggests that the oxidation-reduction process was severely disrupted by garlic oil treatment. Oxidation-reduction processes are ubiquitous and essential to *in vivo* biochemical processes with regulatory functions critical for cell signal transduction and gene transcription. The disruption of oxidation-reduction processes is fatal to cells. Most of the differentially expressed genes related to pathogenesis were downregulated, and a small portion was upregulated. This indicates that garlic oil caused downregulation of pathogenicity-related genes, and in turn could reduce the virulence and pathogenicity of *C. albicans*. The significantly enriched GO term associated with cellular response to starvation suggests that the normal metabolic activity of *C. albicans* cells was also disrupted. Similarly, the significant enrichment of GO terms in the molecular function category indicates that the expression levels of ATP-binding, zinc-ion-binding, and DNA-binding genes were significantly changed. The notably enriched terms in the cellular component category suggest that nearly all cellular components were affected including the nucleus, plasma membrane, cytoplasm, ribosomes, and the cell surface.

The differentially expressed genes were primarily clustered in 19 KEGG pathways. The most significant KEGG pathway identified was associated with oxidative phosphorylation. Oxidative phosphorylation is an important biochemical process in the cell. It is the final metabolic pathway of cellular respiration and a key step required for ATP generation, and occurs in the inner membrane of the mitochondria of eukaryotic cells. Consistent with the finding using KEGG analysis, we observed severe mitochondrial damages in garlic oil-treated *C. albicans* cells. These results indicated that oxidative phosphorylation occurring in the inner membrane of the mitochondria was severely disrupted. The second KEGG pathway identified was associated with spliceosomes. RNA splicing is a very important biological process of gene expression in eukaryotic cells, and protein synthesis is critically dependent upon spliceosome activity. Gene transcripts must undergo RNA splicing in order to become mature mRNA containing appropriate protein coding information, and therefore, RNA splicing is vital for gene expression. Disruption of this pathway indicates that *C. albicans* gene expression was severely affected by garlic oil treatment. Given the effects on this spliceosome pathway, it is not surprising that so many other KEGG pathways were also affected by garlic oil treatment including the cell cycle, protein processing in the endoplasmic reticulum, pyrimidine metabolism, meiosis, RNA transport, ribosome biogenesis, RNA degradation, proteasome functions, the mRNA surveillance pathway, nucleotide excision repair, basal transcription factors, DNA replication, RNA polymerase activity, various types of N-glycan biosynthesis, protein export, and the MAPK signaling pathway. The KEGG pathways of the cell cycle, meiosis, and DNA replication are all closely related to cell reproduction, whereas the pathways for RNA transport, RNA degradation, mRNA surveillance, and RNA polymerase are all responsible for gene expression. Protein processing in the endoplasmic reticulum, ribosome biogenesis in eukaryotes, nucleotide excision repair, and protein export are important KEGG pathways in the regulation of protein synthesis. Additionally, the N-glycan biosynthesis pathways are responsible for glycan biosynthesis. Therefore, almost all of the critical physiological and metabolic processes of *C. albicans* were severely impacted by garlic oil treatment.

The 2D-DIGE results showed the upregulation of putative cytoplasmic adenylate kinase, pyruvate decarboxylase, hexokinase and heat shock protein Ssc1. The putative cytoplasmic adenylate kinase belongs to the adenylate kinase family, and adenylate kinase catalyzes the reversible transfer of the terminal phosphate group between adenosine triphosphate (ATP) and adenosine monophosphate (AMP)^21^,^22^,^23^,^24^. It also plays an important role in cellular energy homeostasis and in the metabolism of adenine nucleotides. Adenylate kinase activity is critical for the regulation of the phosphate utilization and AMP *de novo* biosynthesis pathways^21^,^22^,^23^,^24^. KEGG pathway analysis by RNA sequencing demonstrated that the energy balance in the cells was altered due to the disruption in cellular oxidative phosphorylation. Consistent with the RNA sequencing results, the 2D-DIGE analysis indicates with high probability that cytoplasmic adenylate kinase upregulation may be required to maintain energy homeostasis in the cell.

Pyruvate decarboxylase is a thiamin diphosphate-dependent enzyme within the glycolytic pathway in fermenting cells. It catalyzes the non-oxidative conversion of pyruvate to acetaldehyde and carbon dioxide^25^. Pyruvate decarboxylase and pyruvate dehydrogenase utilize pyruvate as a substrate to produce either ethanol or initiate the citric acid (TCA) cycle. Under anaerobic conditions, the concentration of pyruvate is elevated by glycolysis, and pyruvate decarboxylase catalyzes the generation of ethanol from pyruvate. Under aerobic condition, the concentration of pyruvate is reduced, and pyruvate dehydrogenase catalyzes the generation of acetyl-CoA from pyruvate for subsequent initiation of the TCA cycle^25^. The upregulation of pyruvate decarboxylase shown in the proteomic analysis suggests the presence of anaerobic conditions in the cellular metabolic process. The highly probable elevation of putative cytoplasmic adenylate kinase and pyruvate decarboxylase detected by 2D-DIGE analysis were both consistent with severely disturbed oxidative phosphorylation and cellular respiration pathways revealed by KEGG analysis of RNA sequencing results.

Hexokinase is an intracellular enzyme that catalyzes phosphorylation of glucose, mannose, and fructose into the corresponding hexose 6-phosphates, which could then be broken down into pyruvate by mean of glycolysis, or utilized for various biosynthesis reactions^26^. Upregulation of hexokinase might result from the need for increased glycolysis to supply energy in *C. albicans* cells treated with garlic oil. This is also consistent with the severe oxidative phosphorylation and cellular energy balance disturbances indicated by the KEGG analysis.

The dramatic upregulation of the heat shock proteins is a key part of the heat shock response, induced primarily by heat shock factors^27^. Production of high levels of heat shock proteins may also be triggered by exposure to different types of environmental stress conditions such as cellular exposure to toxins, starvation, hypoxia, or water deprivation^28^. Therefore, heat shock proteins are also referred to as stress proteins, and their upregulation is often described more generally as part of the stress response. The significant upregulation of heat shock protein Ssc1 might be associated with the stress response of *C. albicans* cells as a result of exposure to garlic oil. The GO analysis of the RNA sequencing also demonstrated some degree of cellular response in *C. albicans* similar to that observed in cellular responses to drugs and starvation. Therefore, the results of 2D-DIGE were consistent with that of RNA sequencing.

In conclusion, garlic oil exhibited strong antifungal activity against *C. albicans*, and its MIC was 0.35 μg/mL. Moreover, garlic oil had a time- and dose-dependent antifungal effect on *C. albicans* cells. Garlic oil could penetrate not only the cellular membrane, but also the membranes of organelles such as the mitochondria, resulting in damaged organelles and cell death. In addition, garlic oil treatment induced differential expression of several critical genes including those involved in the cellular drug response, oxidation-reduction processes, pathogenesis, and the cellular starvation response. Moreover, the differentially expressed genes were mainly clustered in 19 KEGG pathways associated with oxidative phosphorylation, spliceosome, cell cycle, protein processing in endoplasmic reticulum, pyrimidine metabolism, meiosis, RNA transport, ribosome biogenesis, and RNA degradation. In addition, four of the upregulated proteins were identified by MS as highly probable for putative cytoplasmic adenylate kinase, pyruvate decarboxylase, hexokinase, and heat shock proteins. These results suggest that anaerobic metabolic processes are involved in some of the responses observed in *C. albicans* cells following garlic oil treatment. Many proteins were downregulated, resulting in significant disruptions of the normal cellular metabolism and physical functions of *C. albicans*.

## Materials and Methods

### Chemical reagents, microorganisms, media, and culture condition

Garlic oil was purchased from Guangzhou Baihua Flavours and Fragrances Company Ltd (Guangzhou, China). The garlic oil was pure with a density of 1.108 g/mL. *C. albicans* strain ATCC 10231 was purchased from the American Type Culture Collection (ATCC) and maintained in our laboratory before experiments. Sabouraud dextrose broth (SDB) medium (purchased from Guangzhou Huankai Microbial Sci. and Tech. Co., Ltd, Guangzhou, China) used for *C. albicans* aerobic cultivation at 28 °C with shaking at 150 rpm contained the following (per liter): 10 g of peptone, 40 g of dextrose, final pH 5.6 ± 0.2. Sabouraud dextrose agar (SDA) medium was prepared by adding 1.5% agar to SDB medium. All solvents and reagents were of analytical grade.

### Measurements of antifungal activity of garlic oil against *C. albicans*

Antifungal activity was measured using the poisoned food technique as described in our previous study^20^ with slight modification. The experimental concentrations of garlic oil were 0 (as control), 0.04, 0.09, 0.17, 0.35 and 0.69 μg/mL. Every SDA plate was inoculated with approximately 10^5^ CFU of *C. albicans*. The plates were sealed with parafilm and incubated at 28 °C in an incubator for 7 days. The MIC of garlic oil against *C. albicans* was determined as the lowest concentration at which no visible growth was observed during the 7 day incubation period. The experiment was carried out in triplicate.

### Fungicidal kinetics of garlic oil against *C. albicans*

The fungicidal kinetics of garlic oil against *C. albicans* was determined as described in our previous study^20^. The experimental concentrations of garlic oil in SDB medium were 0 (as control), 0.04, 0.09, 0.17, 0.35, 0.69, 1.39 and 2.77 μg/mL. The concentrations of *C. albicans* cells in SDB medium were all approximately 10^5^ CFU/mL. Fungicidal kinetics curves were plotted based on the total number of living cells per milliliter, with the number of living cells in log scale on the ordinate and the processing time on the abscissa. The experiment was carried out in triplicate.

### TEM observation of the internal morphology of planktonic *C. albicans* cells exposed to garlic oil

The experimental methods used to evaluate the antifungal effect of garlic oil on *C. albicans* cells were the same as those described in our previous study^29^. The experimental concentrations of garlic oil were 0 (as control) and 1.39 μg/mL. After treatment for 2 h, the cells were sampled and prepared as ultrathin sections, which were observed under a TEM (Hitachi H-7650). The experiment was carried out in triplicate.

### RNA-seq, bioinformatics analysis, and accession numbers

Two separate 50 mL cultures containing SDB medium and *C. albicans* cells were added to two conical flasks. The initial cell concentrations in the cultures were both 10^5 ^CFU/mL. The cultures were incubated in a water bath shaker at 28 °C with shaking at 150 rpm for 12 h. Garlic oil or PBS buffer were then added to the cultures to achieve 0 (control) or 1.39 μg/mL of garlic oil. The cultures were continuously incubated at the same conditions for 5 h. The cells were then sampled and centrifuged. The cell precipitates in the control and the 1.39 μg/mL garlic oil groups were snap frozen at −80 °C separately.

Total RNA was isolated from cells using Trizol (Life Technologies, USA) according to the manufacturer’s protocol. RNA purity was assessed using an ND-1000 NanoDrop. Each RNA sample had an A260: A280 ratio between 1.8 and 2.0. RNA integrity was evaluated using the Agilent 2200 Tape Station (Agilent Technologies, USA), and each sample had an RNA integrity number above 7.0. In brief, mRNAs were isolated from total RNA and fragmented to approximately 200 bps. Subsequently, the collected mRNAs were subjected to first strand and second strand cDNA synthesis, followed by adaptor ligation and low-cycle enrichment according to the instructions of TruSeq RNA LT/HT Sample Prep Kit (Illumina, USA).

The purified library products were evaluated using the Agilent 2200 Tape Station and Qubit 2.0 (Life Technologies, USA). RNA sequencing was performed at Guangzhou RiboBio Co., Ltd. on an Illumina HiSeq 2500. Prior to sequencing, the raw data were filtered to produce high-quality clean data. All the subsequent analyses were performed using the clean data. RNA sequencing data are available in the Gene Expression Omnibus (GEO) database under the accession numbers GSE70524.

The reference genome and gene model annotation files were downloaded directly from the NCBI library. The clean reads were aligned to the reference genome using TopHat (Broad Institute, Cambridge, MA, USA). During read alignment, a maximum of two mismatches and a gap length of 2 bps were allowed. The functional annotations of genetic variants were generated using ANNOVAR^30^. Read alignments were processed by Cufflinks software package^31^ to determine the differential expression of genes. Gene expression values were quantified as reads per kilobase of transcript per million mapped reads (RPKM). Statistical analysis of the differentially expressed genes was performed using DEGseq software.

To better understand the functional implications and the metabolic pathways of the differentially expressed genes, the web-based gene set analysis toolkit WebGestalt^32^,^33^ was employed to retrieve the gene ontology (GO) categories and KEGG pathways associated with the differentially expressed genes. WebGestalt used a hypergeometric test to calculate the *p*-values to evaluate the statistical significance of enrichment within a category by comparing the occurrence of genes in the input against that of the reference database. Then, the *p*-values were adjusted for the multiple tests performed using the Benjamini and Hochberg method^34^. For comparison, the GO categories and KEGG pathways of the differentially expressed genes were also obtained.

### 2D-DIGE proteomic analysis for planktonic *C. albicans* cells exposed to garlic oil

Two separate 100 mL cultures containing SDB medium, *C. albicans* cells and garlic oil were added to conical flasks. The initial cell concentrations in culture were both 10^6 ^CFU/mL, and the concentrations of garlic oil were 0 (control) and 1.39 μg/mL. The cultures were then incubated in a water bath shaker at 28 °C with shaking at 150 rpm for 2 h. The cells were then sampled and centrifuged. The cell precipitates in the control and 1.39 μg/mL garlic oil groups were snap frozen at −80 °C separately.

Cell pellets were successively re-suspended in chilled distilled water and in lysis buffer [7 M urea, 2 M thiourea, 4% CHAPS buffer, 30 mM Tris/HCl (pH 6.8)]. Glass beads and 1 mM PMSF were added to the cell suspension. Then, the mixture was vortexed and subjected to ultrasound. The cell extract was centrifuged and the supernatant was kept as a crude protein sample. The protein samples were purified by precipitation using a 2-D Clean-up kit (GE Healthcare) following the procedure recommended by the manufacturer. The protein concentration was determined via a copper iron assay using a 2-D Quant kit (GE Healthcare)^35^.

2D-DIGE was performed at the Center for Proteomics of the School for Basic Medical Science at Sun Yat-Sen University. Two 2D-analysis gels and two preparative gels were prepared, in which proteins were labeled with CyDye (GE Healthcare). Three fluorescent dyes, Cy2, Cy3 and Cy5, were used to label the samples using a chemical coupling method^36^. The immobilized DryStrip (pH 3–10, GE Healthcare) was embedded in 0.5% (w/v) agarose on top of 12.5% acrylamide gel after rehydration and equilibration. Four gels were performed simultaneously on an Ettan DALTsix electrophoresis system (GE Healthcare). The Cy2 (pool sample)-, Cy3 (control sample)- and Cy5 (1.39 μg/mL garlic oil-treated sample)-labelled proteins of the analysis gels were imaged individually using a DIGE-enabled Typhoon imager 9400 (GE Healthcare). The preparative gels were stained with a Deep Purple total protein stain (GE Healthcare) according to the manufacturer’s instructions^35^.

DeCyder software (GE Healthcare) was used to detect and quantify fluorescence intensity in images. We selected protein spots whose mean expression ratios were greater or less with at least 3-fold. The proteins of interest were excised robotically by an Ettan spot handling workstation (GE Healthcare) for subsequent MS and database interrogation. Peptide mass fingerprinting was carried out by MALDI-TOF-MS (Ettan, GE Healthcare). Proteins identified by peptide mass fingerprinting were interrogated using the MASCOT search engine^35^. The following parameters were used in the MASCOT database query: Peptide Mass Fingerprint (Type of search), Trypsin (Enzyme), Carbamidomethyl (C) (Fixed modifications), Oxidation (M) (Variable modifications), Monoisotopic (Mass values), Unrestricted (Protein Mass), ± 100 ppm (Peptide mass tolerance), 1 (Max missed cleavages).

## Additional Information

**How to cite this article**: Li, W.-R. *et al.* Antifungal activity, kinetics and molecular mechanism of action of garlic oil against *Candida albicans*. *Sci. Rep.* **6**, 22805; doi: 10.1038/srep22805 (2016).

## Figures and Tables

**Table 1:** List of identified *C. albicans* proteins.

Protein no.	Density ratio	Protein description	Mass(Da)	NCBI accession no.
2098	+5.13	putative cytoplasmic adenylate kinase [*Candida albicans* SC5314]	27790	gi|68484570
529	+4.41	pyruvate decarboxylase [*Candida albicans* SC5314]	62744	gi|723212668
716	+3.19	hexokinase [*Candida albicans* SC5314]	53836	gi|723204716
437	+3.13	heat shock protein Ssc1, mitochondrial precursor [*Candida albicans* WO-1]	69802	gi|238883630
2864	−5.33	hypothetical protein CaO19.11218 [*Candida albicans* SC5314]	30899	gi|68485249
2836	−5.22	hypothetical protein CaO19.2924 [*Candida albicans* SC5314]	173080	gi|68481073
2629	−4.48	hypothetical protein CaO19.1102 [*Candida albicans* SC5314]	12615	gi|68492427
2789	−4.37	hypothetical protein CaO19.3622 [*Candida albicans* SC5314]	49636	gi|68483258
2095	−3.50	hypothetical protein CaO19.4311 [*Candida albicans* SC5314]	16937	gi|68470745
